# The complete chloroplast genome of *Camellia fluviatilis* (Theaceae), a wild oil-*Camellia* species

**DOI:** 10.1080/23802359.2021.2005482

**Published:** 2021-11-29

**Authors:** Meiying Yang, Fengyu Xie, Jianbin Li, Ying Zhang, Xinlei Li, Hengfu Yin, Jiyuan Li

**Affiliations:** a Research Institute of Subtropical Forestry, Chinese Academy of Forestry, Hangzhou, China; b College of Agronomy and Biotechnology, Yunnan Agricultural University, Kunming, China; c College of Landscape and Forestry, Qingdao Agricultural University, Qingdao, China

**Keywords:** *Camellia fluviatilis*, chloroplast genome, phylogenetic analysis

## Abstract

*Camellia fluviatilis* is an important shrub producing edible seed oil, which is widely cultivated in South China. In this study, the complete chloroplast genome was sequenced and analyzed based on the Illumina HiSeq platform. The results showed that the complete chloroplast genome is 157,041 bp with 37.29% GC content, including a large single copy (LSC) region of 86,718 bp, a small single copy (SSC) region of 18,293 bp, and a pair of inverted repeats (IRs) regions of 26,015 bp. There are 128 genes in the chloroplast genome of *C. fluviatilis,* including 83 protein-coding genes, 8 ribosomal RNAs, and 37 transfer RNAs. Phylogenetic analysis revealed that *C. fluviatilis* is closely related to *C. lanceoleosa*, indicating that both belong to the Sect. Paracamellia Sealy.

*Camellia fluviatilis* Hand. – Mazz. (1922), a wild species producing high-quality edible oils, is widely cultivated and applied in South China. Currently, *C. fluviatilis* has different classification statuses in Flora Reipublicae Popularis Sinicae (Zhang and Ren 1998) and Monograph of the Genus *Camellia* (Min 2000), and its genomic information is scarce. The chloroplast genome contains the important genetic information to clarify phylogenetic relationships (Liang et al. 2021). In this study, high-throughput sequencing was performed to reveal the assembly and annotation details of the complete chloroplast genome in *C. fluviatilis* (NCBI Accession Number: MT948190).

The specimen of *C. fluviatilis* was deposited at the Research Institute of Subtropical Forestry, Chinese Academy of Forestry (http://risfcaf.caf.ac.cn/; Coordinates: 119°95′E, 30°07′N; Xinlei Li, lixinlei2020@163.com) under the voucher number YL914766. The total genomic DNA of *C. fluviatilis* was extracted by the MiniBEST plant Genomic DNA Extraction Kit (Takara, Dalian, China). The DNA concentration quality of the sequencing sample was higher than 20 ng/µL, and the total mass was higher than 100 ng measured by a NanoDrop 2000 device (Thermo Fisher Scientific, USA). After Illumina sequencing libraries were constructed, the high-throughput sequencing was carried out by the Illumina HiSeq 4000 sequencing system (Illumina, San Diego, California, USA).

In total, 25,206,648 raw reads and 3,655,585,161 raw bases were obtained in the initial stage, then the reads and bases were trimmed with Trimmomatic to obtain 24,456,549 clean reads and 3,501,550,697 clean bases (Bolger et al. 2014; Xu et al. 2018). Clean reads were compared with the reference genome sequence of *C. japonica* (NCBI Accession Number: NC_036830.1) by Bowtie v2.2.6 (Wang et al. 2018; Cao et al. 2020), and the sequence of the genome was assembled by Newbler v3.0 (Ye et al. 2017). Finally, the genome was annotated using package Geseq (Tillich et al. 2017) and corrected manually using Geneious v.9.0.2 (Kearse et al. 2012).

The results revealed 128 functional genes in the genome of *C. fluviatilis*, which contains 83 protein-coding genes (CDs), 37 transfer RNA (tRNA) genes, and 8 ribosomal RNA (rRNA) genes. The complete chloroplast genome assembled is 157,041 bp in length with 37.29% GC, including a large single-copy (LSC) region of 86,718 bp, a small single-copy (SSC) region of 18,293 bp, and a pair of inverted repeats (IRs) of 26,015 bp, displaying the typical quadripartite structure.

To study evolutionary relationships, the phylogenetic tree of *Camellia* was reconstructed based on the Neighbor-joining (NJ) analysis of concatenated chloroplast protein-coding genes sequences for sequenced chloroplast genomes. The conserved protein sequences were performed for the alignment (Wang et al. 2018), and the phylogenetic tree was reconstructed by MEGA v7.0.14 (Kumar et al. 2016). *Schima superba*, the species of genus *Schima*, Theaceae was used as the outgroup. According to the classification system of Flora Reipublicae Popularis Sinicae, *C. fluviatilis* belongs to the Sect. Paracamellia Sealy and *C. lanceoleosa* belong to the Sect. Oleifera Sealy (Zhang and Ren 1998). However, *C. fluviatilis* and *C. lanceoleosa* were divided into the Sect. Paracamellia Sealy and *C. lanceoleosa* were classified as the variety of *C. fluviatilis* in the classification system of Monograph of the Genus *Camellia* (Min 2000). It was shown that *C. fluviatilis* was closely related to *C. lanceoleosa* based on the phylogenetic tree (Figure 1), indicating that the results of the present study support the classification system of Monograph of the Genus *Camellia* (Min 2000).

**Figure 1.**
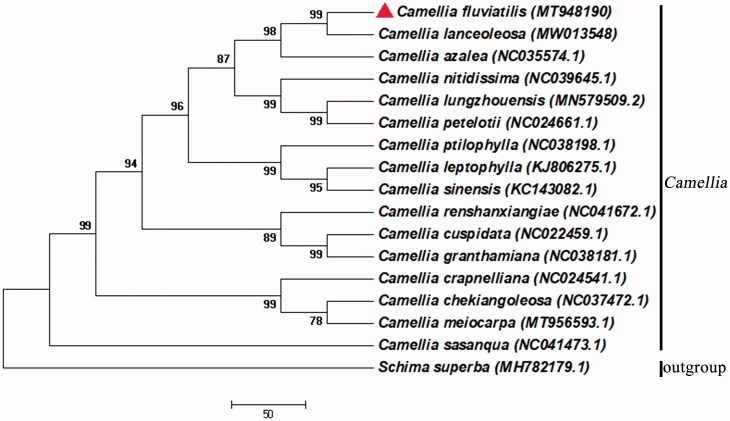
The neighbor-joining phylogenetic tree for *C. fluviatilis* with other *Camellia* species based on conserved protein sequences of the complete chloroplast genomes by MEGA v7.0.14. The bootstrap support values of >50% from 1000 replicates are listed for each node.

## Data Availability

The genome sequence data that support the findings of this study are openly available in GenBank of NCBI at [https://www.ncbi.nlm.nih.gov] under the accession No. MT948190. The associated BioProject, SRA, and Bio-Sample numbers are PRJNA744037, SRR15050580, and SAMN20066001 respectively.
